# Universal Viral Screening of Patients with Newly Diagnosed Cancer in the United States: A Cost-efficiency Evaluation

**DOI:** 10.1158/2767-9764.CRC-23-0255

**Published:** 2023-09-28

**Authors:** Riha Vaidya, Joseph M. Unger, Rohit Loomba, Jessica P. Hwang, Rashmi Chugh, Monica A. Tincopa, Kathryn B. Arnold, Dawn L. Hershman, Scott D. Ramsey

**Affiliations:** 1 Fred Hutchinson Cancer Center, Seattle, Washington.; 2 SWOG Statistics and Data Management Center, Seattle, Washington.; 3 University of California San Diego, Moores Cancer Center, San Diego, California.; 4 The University of Texas, MD Anderson Cancer Center, Houston, Texas.; 5 University of Michigan, Rogel Cancer Center, Ann Arbor, Michigan.; 6 UCSD Health, San Diego, California.; 7 Columbia University, New York, New York.

## Abstract

Recommendations for universal screening of patients with cancer for hepatitis B virus (HBV), hepatitis C virus (HCV), and human immunodeficiency virus (HIV) are inconsistent. A recent multisite screening study (S1204) from the SWOG Cancer Research Network found that a substantial number of patients with newly diagnosed cancer had previously unknown viral infections. The objective of this study was to determine the cost-efficiency of universal screening of patients with newly diagnosed cancer. We estimated the cost-efficiency of universal screening of new cancer cases for HBV, HCV, or HIV, expressed as cost per virus detected, from the health care payer perspective. The prevalence of each virus among this cohort was derived from S1204. Direct medical expenditures included costs associated with laboratory screening tests. Costs per case detected were estimated for each screening strategy. Secondary analysis examined the cost-efficiency of screening patients whose viral status at cancer diagnosis was unknown. Among the possible options for universal screening, screening for HBV alone ($581), HCV alone ($782), HBV and HCV ($631) and HBV, HCV, and HIV ($841) were most efficient in terms of cost per case detected. When screening was restricted to patients with unknown viral status, screening for HBV alone ($684), HBV and HCV ($872), HBV and HIV ($1,157), and all three viruses ($1,291) were most efficient in terms of cost per newly detected case. Efficient viral testing strategies represent a relatively modest addition to the overall cost of managing a patient with cancer. Screening for HBV, HCV, and HIV infections may be reasonable from both a budget and clinical standpoint.

**Significance:**

Screening patients with cancer for HBV, HCV, and HIV is inconsistent in clinical practice despite national recommendations and known risks of complications from viral infection. Our study shows that while costs of viral screening strategies vary by choice of tests, they present a modest addition to the cost of managing a patient with cancer.

## Introduction

Viral infections pose risks in patients with cancer, both due to the potential for viral reactivation or exacerbation related to receipt of cancer therapy and due to long-term consequences of harboring the virus in cancer survivors. A recent multisite screening study from the SWOG Cancer Research Network found that a substantial number of patients with newly diagnosed cancer with viral infections, particularly hepatitis B virus (HBV) and hepatitis C virus (HCV), were unaware of their viral status prior to presentation to oncology clinics (1).

Despite national screening recommendations for HBV (2–4), HCV (5, 6), and human immunodeficiency virus (HIV; refs. 7, 8), screening individuals for these viruses is inconsistent in primary care practice (9–11); as such, patients are sometimes referred to oncology practices with undetected viral infection. In 2020, the American Society of Clinical Oncology (ASCO) updated their guidance to recommend that all patients with newly diagnosed cancer be tested for HBV (12). Similarly, the European Conference on Infections in Leukemia (13) and the American Society for Blood and Marrow Transplantation Task Force (14) both recommend all patients with hematologic malignancies be screened for hepatotropic viruses before treatment (15). The National Comprehensive Cancer Network (NCCN) recommends universal HBV, HCV, and HIV (16) screening in patients with cancer expected to receive chemotherapy or immunosuppressive therapy (17).

There are several arguments in favor of universal viral screening of patients with cancer (18). The rate of HBV reactivation has been reported to be as high as 70% among HBsAg-positive individuals receiving standard chemotherapy, leading to serious morbidity and even mortality (15, 19–23). Recent studies suggest that prophylactic antiviral therapy can prevent complications of HBV and HCV during chemotherapy (18). Immunosuppression associated with chemotherapy can also adversely affect HIV-infected people, leading to an elevated risk of infections. HIV-infected individuals may also be more susceptible than uninfected individuals to myelosuppression (24, 25). These issues are increasingly relevant given the move to provide greater access to cancer trials for HIV-positive patients (26). Treatments for HCV and HIV have improved dramatically over time, thus cancer survivors with viruses that are undiagnosed or untreated miss an opportunity to receive care that may substantially reduce their lifetime risk of morbidity and mortality related to infection.

While viral screening tests themselves are inexpensive, given the number of patients with newly diagnosed cancer, it is important to consider system-wide costs when developing viral screening strategies. Multiple studies have evaluated the cost-effectiveness of screening patients with cancer for HBV infection (27–29), evidence on cost-effectiveness is mixed and dependent on risk of HBV reactivation (29) and tests used (27). Moreover, there is little research examining costs associated with screening patients with cancer for multiple viral infections or estimating the total costs of such a screening effort. An estimated 1.9 million patients were diagnosed with cancer in 2022 (30). A significant fraction will receive systemic anticancer therapies. The benefits of screening programs must be balanced against the cost and yield of those programs.

Accordingly, the objective of this study was to determine the most efficient approach to universal screening of patients with newly diagnosed cancer for HBV, HCV, and/or HIV.

## Materials and Methods

### Data and Base Case

We considered a population of persons 18 years or older and presenting for evaluation or treatment of a malignant neoplasm at an oncology practice, including patients presenting for second opinions of confirmed new malignant neoplasms. The prevalence of each virus among this cohort was obtained from SWOG S1204 (ClinicalTrials.gov identifier: NCT01946516; ref. 1). This multicenter, prospective study evaluated the prevalence of latent HBV, HCV, and HIV infection among patients ages ≥18 years with a newly diagnosed cancer (including hematologic). Among the 3,051 patients with newly diagnosed cancer enrolled in the study, the estimated U.S. viral infection rates for previous HBV, chronic HBV, HCV, and HIV were 5.3%, 0.4%, 1.9%, and 1.0%, respectively. These rates were considered nationally representative because the study analysis adjusted for distributional differences in the study population compared with the U.S. cancer population with respect to type of cancer, age (<65 vs. ≥65 years) and race (White vs. non-White) using data from the Surveillance, Epidemiology, and End Results Program. These prevalence estimates included individuals newly diagnosed with HBV, HCV, or HIV infection as well as those who were previously diagnosed with these infections. Direct medical expenditures were estimated using costs associated with laboratory screening tests for each virus, using tests recommended by CDC and USPSTF screening guidelines (31–34). The HBV screening tests included a surface antigen test (HBsAg, CPT 87340), a surface antibody test (anti-HBs, CPT 86706), and a core antibody test (anti-HBc, CPT 86704). Costs were sourced from the 2022 CMS Laboratory Test Reimbursement Schedule (35). Detection of HCV was done via an antibody test (CPT 86803) that, when positive, was followed by a nucleic acid test (CPT 87520). Screening for HIV entailed a fourth-generation immunoassay that, when positive, was followed by an HIV-1/HIV-2 differentiation test (CPT 86701/86702) as detailed in Table 1.

**TABLE 1:** Model input parameters

Parameter	Base case	Low	High
Overall prevalence^a^ – Base case analysis			
HIV	1.0%	0.6%	1.4%
HCV	1.9%	1.4%	2.4%
Chronic HBV	0.4%	0.2%	0.6%
Past HBV	5.3%	4.5%	6.1%
Prevalence of known infection – Secondary analysis			
HIV	0.9%	0.6%	1.3%
HCV	1.3%	0.9%	1.7%
Chronic HBV	0.2%	0.1%	0.4%
Past HBV	0.7%	0.4%	1.0%
Prevalence of unknown infection – Secondary analysis			
HIV	0.1%	0.0%	0.2%
HCV	0.6%	0.3%	0.9%
Chronic HBV	0.2%	0.0%	0.3%
Past HBV	4.7%	3.9%	5.5%
Costs^b^			
Fourth-generation immunoassay for HIV (EIA/ELISA) (CPT 87389)	$24.08	$12.04	$48.16
HIV-1/HIV-2 differentiation test (CPT 86701/86702)	$22.41	$11.21	$44.82
HCV antibody test (CPT 86803)	$14.27	$7.14	$28.54
HCV nucleic acid test (CPT 87520)	$31.22	$15.61	$62.44
Hepatitis B surface antigen test (CPT 87340)	$10.33	$5.17	$20.66
Hepatitis B core antibody test (CPT 86704)	$12.05	$6.03	$24.10
Hepatitis B surface antibody test (CPT 86706)	$10.74	$5.37	$21.48

Abbreviations: CPT, current procedural terminology; EIA, enzyme immunoassay; ELISA, enzyme-linked immunosorbent assay.

^a^Prevalence estimates obtained from published data for S1204 (Ramsey, et al. 2019)(1).

^b^Test costs from CMS Diagnostic Laboratory Fee Schedule. Accessed from https://www.cms.gov/medicaremedicare-fee-service-paymentclinicallabfeeschedclinical-laboratory-fee-schedule-files/22clabq4 (35).

We identified seven screening strategies—screening for each virus individually (HBV alone, HCV alone, HIV alone), screening for two viruses (HBV and HCV, HBV and HIV, HCV and HIV), and screening for all three viruses. For each screening strategy, we estimated costs per 1,000 patients screened and costs per case detected. For each individual viral screening, the cost of screening 1,000 patients for the virus was calculated using the formulas in equations (1) to (3), with C indicating the cost of the test. For screening strategies that screened for two or three viruses, cost was calculated as the sum of screening costs (per 1,000 patients) for each viral screening included in the strategy. Cost per case detected was calculated as screening cost divided by the number of cases detected, where the number of cases was obtained on the basis of the nationally representative prevalence rates from S1204. Costs were analyzed in 2022 USD, and the analysis was performed from a U.S. health care payer perspective.



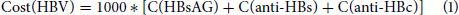





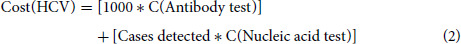





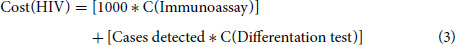



We estimated the annual cost of applying the screening strategies nationally by extrapolating the results to all estimated 1.9 million new cancer cases (30) and to the subset of patients expected to receive systemic anticancer therapy. Approximately 55% of cancer cases in high income countries in 2018 were estimated to have been treated with chemotherapy or targeted therapy (36). We assumed a slightly higher proportion in our calculations (60% of cancer cases) to account for systemic therapies not included in the published estimates.

### Uncertainty Analyses

We quantified the impact of test price and viral prevalence on results for each screening strategy using univariate sensitivity analysis by individually varying parameters and then ranking them by relative impact. We varied each fixed parameter around the maximum and minimum of its uncertainty intervals (95% confidence intervals derived from SEs for epidemiologic characteristics and plausible ranges for costs) and plotted the model results.

### Secondary Analysis

In secondary analysis, we examined the cost per virus detected on the basis of testing patients with unknown viral status only. This analysis was based on the pragmatic approach followed in S1204 where patients enrolled in the study were allowed to submit documentation of viral status (e.g., test results, viral load) in lieu of viral testing if infection status was known. Input parameters for national estimates of unknown viral prevalence were calculated from S1204 as the nationally representative estimates for each virus, multiplied by the proportion with unknown cases. The total cost of each individual viral screening test per 1,000 patients was calculated as indicated in equations (4) to (6).



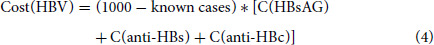





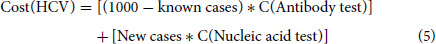





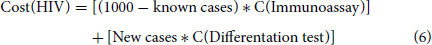



### Data Availability Statement

The data generated in this study are available within the article. Raw data are available upon reasonable request from the corresponding author.

## Results

### Base Case

Among the strategies that screen for a single virus, screening for HBV alone had the lowest cost ($581 per case detected), followed by HCV alone ($782 per case detected). Screening for HIV alone was the most expensive strategy ($2,430 per case detected). Among strategies screening for two viruses, screening for HBV and HCV was the lowest cost option ($631 per case detected). Compared with this strategy, screening for HBV and HIV ($857 per case detected) detected fewer cases for a higher total cost (Table 2). Screening for HCV and HIV had the highest cost per case detected ($1,351) of the strategies screening for two viruses. Screening for all three viruses yielded the most cases detected per 1,000 individuals screened (Table 2), although the total cost of screening was also highest for this strategy. The cost per case detected was $841 when screening for all three viral viruses.

**TABLE 2:** Base case results

Virus	Number of cases detected per 1,000 screened individuals	Cost per 1,000 individuals screened	Cost per case detected
HIV	10	$24,304	$2,430
HCV	19	$14,863	$782
HBV	57	$33,120	$581
HBV and HIV	67	$57,424	$857
HCV and HIV	29	$39,167	$1,351
HBV and HCV	76	$47,983	$631
HBV, HCV, and HIV	86	$72,287	$841

### Uncertainty Analyses

**FIGURE 1:**
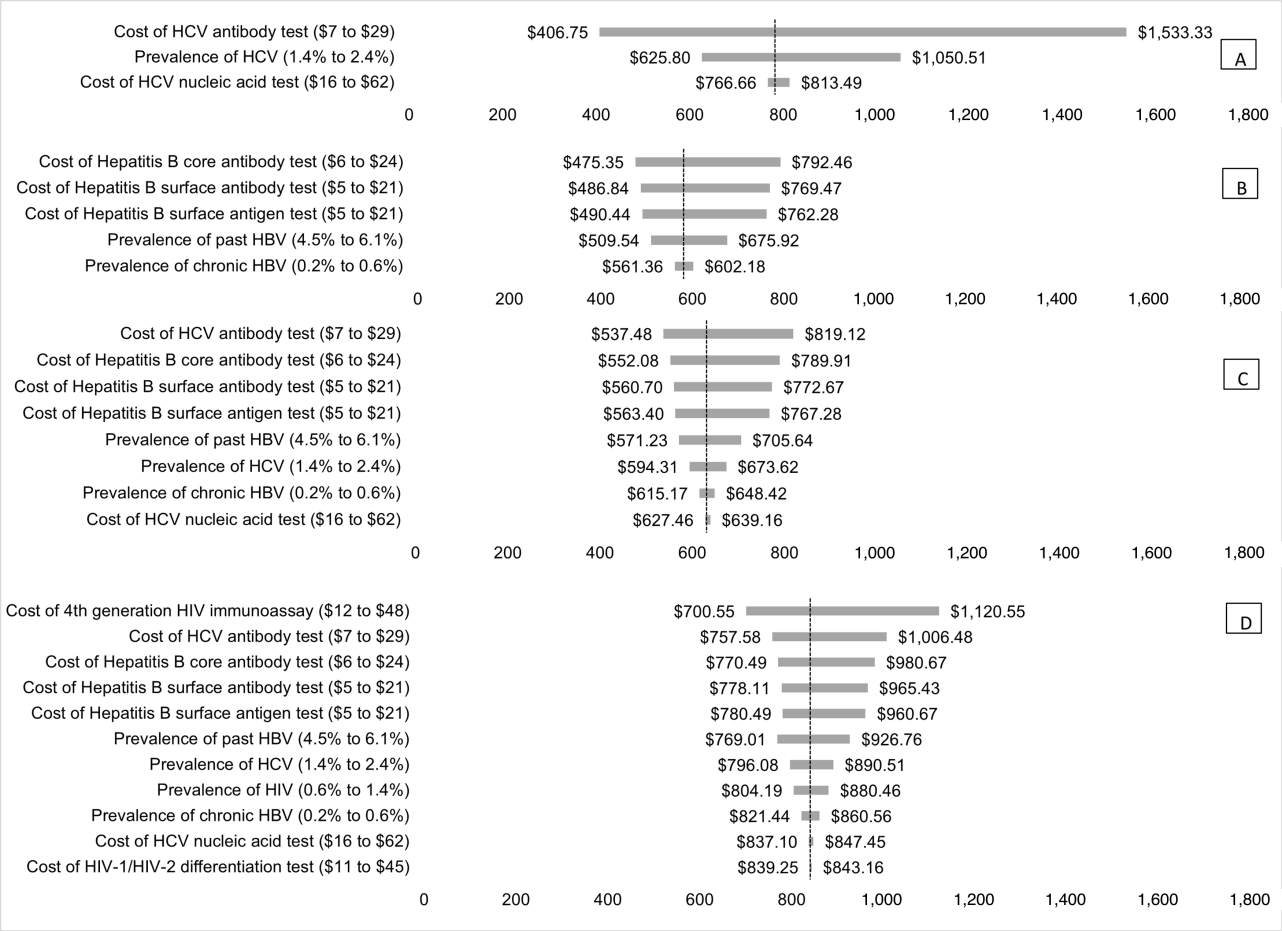
Tornado diagram of most influential inputs for cost per case detected of screening for HCV alone (**A**), HBV alone (**B**), HBV and HCV (**C**), and HBV, HCV, and HIV together (**D**). For each of the four most efficient screening strategies, the figure displays the results from a one-way sensitivity analysis that examines the impact of changing the value of one input at a time on the cost per case detected for the screening strategy. The vertical axis indicates the cost per case detected in the base case scenario for each strategy.

Uncertainty analyses for screening strategies with the lowest cost per case detected are presented in Fig. 1. In univariate uncertainty analyses, the most influential factor for HCV screening was the cost of the HCV antibody test, followed by the prevalence of HCV and the cost of the HCV nucleic acid test (Fig. 1A). The most influential factor for HBV screening was the cost of the HBV core antibody test, followed by the cost of the surface antibody and surface antigen tests (Fig. 1B). The most influential factor for joint HBV and HCV screening were the costs of the HCV antibody test, HBV core antibody test, HBV surface antibody test, and HBV surface antigen test (Fig. 1C). The cost per case detected when screening for all three viruses together was most influenced by the costs of the HIV immunoassay, HCV antibody test, HBV core antibody test, HBV surface antibody test, and HBV surface antigen test (Fig. 1D).

### Estimating National Cost Implications of Screening

Screening all 1.9 million patients with newly diagnosed cancer in the United States for HBV alone, HCV alone, HBV and HCV, and all three viruses would cost approximately $62.9 million, $28.2 million, $91.2 million, and $137.3 million, respectively. These strategies would result in a total of 108,300, 36,100, 144,400, and 163,400 viral infections detected.

Limiting screening to the 1.14 million patients with newly diagnosed cancer who are expected to receive systemic anticancer therapy, the estimated annual screening cost would be approximately $37.8 million, $16.9 million, $54.7 million, and $82.4 million for HBV alone, HCV alone, HBV and HCV, and all three viruses, respectively.

### Secondary Analysis

On the basis of a pragmatic approach that only tests patients with unknown viral status, screening for HBV alone had the lowest cost per newly detected case ($684) among strategies screening for a single virus. Among strategies screening for two viruses, screening for HBV and HCV was least expensive ($872 per newly detected case). Screening for all three viruses led to the largest number of newly detected cases at the highest total cost (Table 3), similar to the base case analysis. However, this strategy had a lower cost per newly detected case ($1,291) compared with screening for HCV alone ($2,379), HCV and HIV ($5,451), and HIV alone ($23,886). Applying this pragmatic screening approach to the 1.9 million cancer cases in the United States, screening for HBV alone, HBV and HCV, HBV and HIV, and all three viruses would result in the detection of 91,200, 102,600, 93,100, and 104,500 previously unknown viral infections, respectively. The total costs associated with these strategies would be approximately $62.4 million, $89.5 million, $107.7 million, and $134.9 million, respectively.

**TABLE 3:** Pragmatic screening approach results

	Number of cases per 1,000 screened individuals			Cost per case	
Virus	Previously known	Newly diagnosed	Cost per 1,000 individuals screened	Per newly diagnosed case	Per identified case (known + unknown)
HIV	9	1	$23,886	$23,886	$2,389
HCV	13	6	$14,272	$2,379	$751
HBV	9	48	$32,822	$684	$576
HBV and HIV	18	49	$56,708	$1,157	$846
HCV and HIV	22	7	$38,158	$5,451	$1,316
HBV and HCV	22	54	$47,094	$872	$620
HBV, HCV, and HIV	31	55	$70,979	$1,291	$825

If this screening approach were only applied to the 1.14 million patients with newly diagnosed cancer who are expected to receive systemic anti-cancer therapy, the estimated annual screening cost would be approximately $37.4 million, $53.7 million, $64.6 million, and $80.9 million for HBV alone, HBV and HCV, HBV and HIV, and all three viruses, respectively.

## Discussion

Patients with cancer who harbor latent viruses while receiving systemic anticancer therapy are at risk for viral reactivation or exacerbation and associated outcomes. Because screening for latent viral infection may be incomplete in primary care, and the prevalence of asymptomatic carriers is significant, screening new patients with cancer is a reasonable option. In this study, we evaluated the cost-efficiency of screening all patients for HBV, HCV, and HIV among patients with newly diagnosed cancer who are planning to receive systemic therapy. Our findings indicated that four strategies (screening for HBV alone, HCV alone, HBV and HCV together, or HBV, HCV, and HIV together) would be the most efficient options for detecting viral infection cases. Indeed, screening for all three viruses together would yield the most cases per person screened. The cost per case detected ranges from $581 (HBV alone) to $841 (HBV, HCV, and HIV together). These represent relatively modest additions to the overall cost of initial care for a patient with cancer which, on average, is estimated to be $41,800 (37). Thus, testing for all three viruses would mean approximately a 2.0% increase in average cost of initial care. We suggest that screening for HBV, HCV, and HIV infections is reasonable from both a cost and clinical standpoint.

Our national cost estimates for screening all newly diagnosed patients with cancer represent the upper bounds of screening costs. Targeted screening programs for high-risk individuals would reduce costs, particularly those that focus on those for whom screening offers little chance of benefit; for example, persons presenting with advanced cancers who forgo systemic anticancer treatment as part of their palliative care management. It is also likely that some patients would refuse the opportunity to be screened. Our estimates of national screening costs for the subset of patients receiving systemic anticancer therapy illustrate the reduced cost burden of alternative screening programs. However, the strategy of excluding patients with reportedly known virus status from screening resulted in very limited overall costs savings (<3% nationally). Because “known” viral status may be at risk of misclassification, this suggests that a universal screening approach may be easier to implement, and more robust. In particular, targeted screening may require additional procedures and possibly costs and may pose risks for adherence. These factors should be carefully considered by oncology clinics, particularly in the context of their resource availability and patient population, when implementing a viral screening program.

Three of the four cost-efficient screening strategies identified in our base case analysis included screening for HBV, while all four cost-efficient strategies in our pragmatic screening-based analysis included screening for HBV. This is aligned with the 2020 ASCO provisional clinical opinion calling for universal HBV screening and systematic management (12). Moreover, a large proportion of HBV infections identified are likely to be previously unknown (1). Thus, a strategy that includes routine screening for HBV is likely to be clinically beneficial. Three of the four strategies support universal HCV screening in patients with cancer, and this is aligned with the NCCN recommendations (17). Screening for HIV, included in the most comprehensive screening strategy of the efficient screening strategies, is aligned with the national universal HIV screening recommendations (8).The majority of cases of HCV and HIV infections will likely be known to patients (1) and adoption of screening practices for these viruses may require a more targeted approach.

Screening for HBV, HCV, and HIV universally at the beginning of the anticancer therapy period would ensure that the underlying viral status is known so as to avoid preventable adverse clinical outcomes. It might also be more efficient for providers than a risk-based approach that would require administration of a questionnaire about risk factors for infection. Our findings present multiple screening strategies that are optimal based on test costs and viral prevalence estimates. Practices that adopt universal viral screening programs may encounter other costs not accounted for in this analysis. Following detection of a viral infection based on screening, practices would need to determine a strategy for prophylaxis or treatment related to viral reactivation as well as modifications to planned cancer treatment. Systems would need to be established to manage patients who are found to harbor active viral infections; for example, referrals to specialty services that treat hepatitis or HIV. Relative to the cost of initial evaluation for newly diagnosed cancer, the cost of viral screening tests will be quite modest and unlikely to be subject to coverage review; however, we feel it is important to establish nationally that payers are willing to cover these screening tests. Planned chemotherapy might need to be delayed or modified until the viral infection is treated, particularly for those whose therapies include anti-CD20 mAbs and/or glucocorticoids (38).

Our findings may have policy implications for payers. One is the issue of insurance coverage for screening among patients with newly diagnosed cancer. USPSTF guidelines support population-wide screening for HCV and HIV (35); thus, for persons who have not had these tests at the time of their presentation to an oncology practice, there are no barriers to reimbursement. In the early phases of S1204, some providers expressed concern about payment for viral screening and the potential cost burden on patients from uncovered tests. In practice, no such issues were encountered (39).

We note the limitations of this study. First, we do not estimate downstream costs and benefits following screening which are likely to differ for each virus. Persons who are found to harbor latent viruses may receive curative viral therapy, thereby altering or delaying anticancer therapy. The costs of managing morbidity and mortality related to hepatitis B reactivation are not estimated, nor are health effects such as prolongation in quality and quantity of life related to eliminating latent viruses. Because there are no reliable data to estimate these impacts, estimating the cost per year of life or quality adjusted year of life (QALY) gained remains speculative and is outside the scope of our analysis. It would be highly valuable for systems that adopt universal screening practices to track outcomes for their patients over an extended time period, such that cost per QALY estimates can be generated for use in the selection of the most cost-efficient screening strategies. Second, our estimates rely on data from a single, multisite study (S1204), albeit the largest study of its kind conducted in the United States. Although the study estimates were adjusted to better reflect the U.S. cancer population, differences between our estimates and the true underlying prevalence of viral infection may exist. Third, our analysis does not account for the downstream costs of unintended transmission of these viruses, particularly to health care workers where there is a known transmission risk. Fourth, our analysis does not account for individual risk factors for viral infection or specific cancer treatment regimens. While current NCCN guidelines recommend viral screening for all patients receiving chemotherapy or immunosuppressive therapy, incorporation of specific risk factors may result in a different set of cost-efficient viral screening strategies. Finally, our study assumes universal adherence to screening and uniformity of laboratory quality standards, although nonadherence would only impact the cost per case detected if it was correlated with likelihood of undiagnosed infection. In this way, the results represent a best-case estimate of the efficiency of screening for HBV, HCV, and HIV.

In summary, using prospectively collected data from a national representative cohort of patients with newly diagnosed cancer, we found that screening for HCV, HBV, and HIV vary widely in terms of costs and cases detected depending on the tests chosen. Further studies are necessary to evaluate the impact of these strategies on cancer morbidity and related lifetime costs.
